# Evaluation of PD-L1 expression on vortex-isolated circulating tumor cells in metastatic lung cancer

**DOI:** 10.1038/s41598-018-19245-w

**Published:** 2018-02-07

**Authors:** Manjima Dhar, Jessica Wong, James Che, Melissa Matsumoto, Tristan Grogan, David Elashoff, Edward B. Garon, Jonathan W. Goldman, Elodie Sollier Christen, Dino Di Carlo, Rajan P. Kulkarni

**Affiliations:** 1 Department of Bioengineering, University of California, Los Angeles, California, USA; 2 Department of Medicine, David Geffen School of Medicine at UCLA, Los Angeles, California, USA; 3 Vortex Biosciences Inc., Menlo Park, California, USA; 4 California NanoSystems Institute, California, USA; 5 Jonsson Comprehensive Cancer Center, California, USA

## Abstract

Metastatic non-small cell lung cancer (NSCLC) is a highly fatal and immunogenic malignancy. Although the immune system is known to recognize these tumor cells, one mechanism by which NSCLC can evade the immune system is via overexpression of programmed cell death ligand 1 (PD-L1). Recent clinical trials of PD-1 and PD-L1 inhibitors have returned promising clinical responses. Important for personalizing therapy, patients with higher intensity staining for PD-L1 on tumor biopsies responded better. Thus, there has been interest in using PD-L1 tumor expression as a criterion for patient selection. Currently available methods of screening involve invasive tumor biopsy, followed by histological grading of PD-L1 levels. Biopsies have a high risk of complications, and only allow sampling from limited tumor sections, which may not reflect overall tumor heterogeneity. Circulating tumor cell (CTC) PD-L1 levels could aid in screening patients, and could supplement tissue PD-L1 biopsy results by testing PD-L1 expression from disseminated tumor sites. Towards establishing CTCs as a screening tool, we developed a protocol to isolate CTCs at high purity and immunostain for PD-L1. Monitoring of PD-L1 expression on CTCs could be an additional biomarker for precision medicine that may help in determining response to immunotherapies.

## Introduction

The immune response to cancer involves a complex network of cellular interactions. Antigen presenting cells (APCs) can recognize neoantigens from some immunogenic tumors^1,2^. These APCs help activate cytotoxic T-cells, helper T-cells and natural killer cells. All of these components work in concert against tumor cells. However, many metastatic tumors such as in non-small cell lung cancer (NSCLC) have adopted methods to evade immune detection and/or clearance^3^. One of the recently discovered pathways that tumors use is the overexpression of programmed cell death ligand 1 (PD-L1). PD-L1 binds to PD-1 on T-cells and suppresses its activity^4,5^. Immunotherapy based on inhibition of PD-1 or PD-L1 represents a breakthrough in the treatment of advanced cancers, particularly lung, renal, and melanoma cancers. Although only a minority of patients have clinical response, those that do often have a durable and lasting response^6–12^.

Finding biomarker predictors of response to PD-L1 blockade has proven challenging. Some studies have suggested that the level of PD-L1 expression on the initial tumor tissue can predict positive response, while other studies suggest that the level of infiltrating CD8+ lymphocytes are the most significant predictors of response^7,10,13^. Past studies of renal cancer show tumors with high PD-L1 levels respond, while those with low levels do not respond^10^. Based on these findings, a clinical trial for anti-PD1 (pembrolizumab) was conducted where NSCLC patients were screened based on expression levels of PD-L1 on the primary tumor^7,14^. Response rate, progression free survival and overall survival with PD-1 inhibitors were greater in tumors with high tumor PD-L1 expression^7,11^. Although current guidelines call for anti-PD1 therapies after failure of standard chemotherapy, these are rapidly changing and recent phase I trial data suggests that anti-PD1 therapies may be effective as first line therapy^15^. First line therapy is especially promising for patients with higher PD-L1 expression on their biopsies and is associated with improved responses^7^.

**Figure 1:**
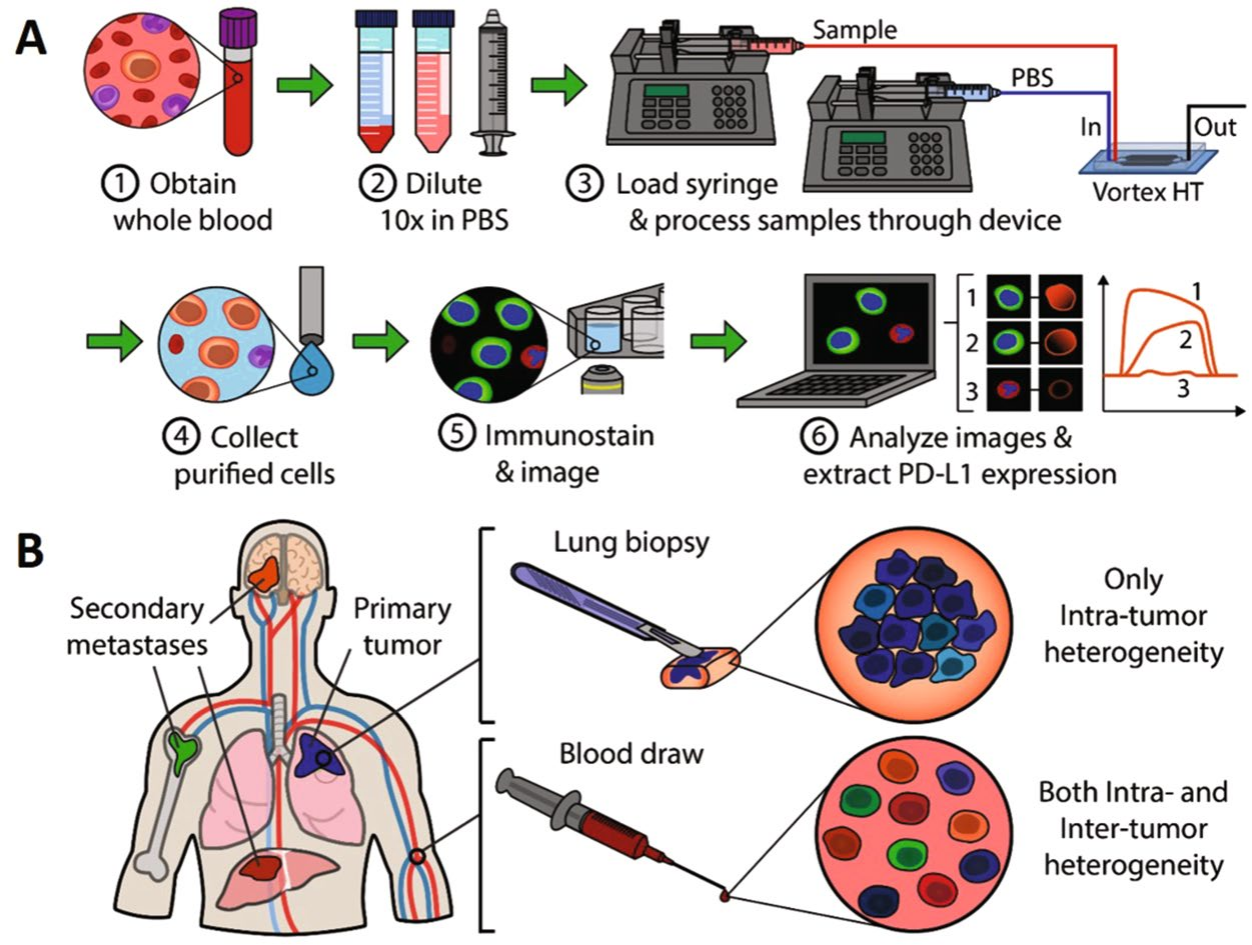
Workflow for evaluation of PD-L1 expression on patient CTC and matched tumor biopsy. (**A**) CTC Workflow. ① Blood is collected from cancer patients and processed through Vortex technology to enrich for CTCs. ② Blood is diluted 10X with PBS and ③ injected through the microfluidic device with syringe pumps. ④ Purified cells are collected into a 96 well-plate, where they are ⑤ stained with immunofluorescence markers and imaged. ⑥ Fluorescence intensity can be analyzed and PD-L1 gene expression quantified. (**B**) Tumor biopsy workflow: In parallel of the CTC workflow, lung biopsies were analyzed for PD-L1 expression. While biopsy provides information on the intra-tumor heterogeneity, only the CTCs present in a blood draw can cover both intra and inter-tumor heterogeneity.

Several challenges exist in screening patients with only an invasive biopsy of the primary tumor. Biopsies allow sampling from limited sections of the tumor at one time point, which may not detect tumor heterogeneity (Fig. 1). Furthermore, especially for lung cancer, the biopsy tissue may be limited or may have been taken much earlier in the cancer’s course (i.e. before it became metastatic). This is because repeat biopsies are avoided due to potential serious complications. If a biopsy is limited to the primary tumor at a single time point, it also does not allow evaluation of other metastasized tumor sites, and the primary tumor may not necessarily be representative of the metastatic sites. As reported, some patients whose primary tumor was negative for PD-L1 still responded well to anti PD-1 treatment, potentially because the biopsy may not have captured the heterogeneous expression of PD-L1 on the tumor^7^. Biopsy of multiple sites or serial biopsies during treatment could address some of these issues, however it may not be feasible due to the invasiveness of the procedure and the potential risks to the patient. In this regard, PD-L1 expression on circulating tumor cells (CTCs) could aid in screening and monitoring patients^16^. CTCs are tumor cells that are shed from various locations of the primary and/or metastatic tumors^17–19^. As such, they may represent a greater portion of the spectrum of genetic and epigenetic variability within a patient’s tumors (Fig. 1). Additionally, monitoring PD-L1 levels over time on CTCs may potentially yield information about modulation of tumor PD-L1 expression in the presence of inhibition of the PD-1/PD-L1 interaction.

There have been few studies to explore PD-L1 expression on CTCs, either in breast cancer (15) or in bladder cancer^20^ and another examining nuclear PD-L1 expression in colon and prostate CTCs^16,21^. To our knowledge, only one recent study from Nicolazzo *et al*. evaluated PD-L1 expression in NSCLC CTCs and examined PD-L1 expression in the context of active immunotherapy treatment, particularly PD-1/PD-L1 inhibition (nivolumab in their study)^22^. Most of these previous studies utilized specific surface markers for CTC capture, either with CellSearch or with similar magnetic bead technology, and did not isolate cells in a manner that is unbiased to surface expression. There are still other knowledge gaps, particularly in how PD-L1 expression on CTCs correlates with expression on tumor biopsies, what method to use for quantifying PD-L1 expression, on both CTCs and tumor biopsies, and how PD-L1 expression on CTCs varies, both at time of initial treatment and as therapy continues.

Here, we evaluate the PD-L1 expression on 31 CTC-containing samples, obtained from 22 patients with metastatic NSCLC who were scheduled to receive or were receiving PD-1 or PD-L1 inhibitors, including 11 metastatic NSCLC patients scheduled to receive the anti-PD-1 treatment pembrolizumab (one patient ended up receiving erlotinib) (Fig. 1A, Table 1). Most patients were evaluated for CTC collection prior to treatment or at the beginning of the second cycle of treatment. For patients receiving pembrolizumab or erlotinib, we compared the quantitative expression of PD-L1 to levels on tumor biopsies taken before treatment when available (N = 4, Fig. 1B) and assessed whether levels were associated with progression free survival (PFS). Tumors of patients receiving pembrolizumab were originally graded as positive for PD-L1 as this was one of the initial inclusion criteria for receiving this therapy.

**Table 1:** Patient cohort, clinical information, and cell enumeration.

No.	Sex	Age	Type	Treatment	Time	Biopsy?	CTCs	Vol.	CTCs/mL	(PDL1 + )	(PDL1−)	(UD)	WBCs/mL
P01	M	61	squamous	Pembrolizumab	Pre	Y	47	6	7.83	4.83	2.83	0.16	60
P02	M	59	adeno.	Pembrolizumab	Pre	—	35	6	5.83	5	0.83	—	6
P03	F	77	adeno.	Ipilimumab, Nivolumab	Pre	—	44	8	5.5	0	5.5	—	17
P04	M	67	adeno.	Nivolumab	Pre	—	28	6	4.67	2.83	1.83	—	56
P05	F	59	adeno.	Pembrolizumab	Pre	Y	19	6	3.17	1.5	1.66	—	18
P06	F	61	adeno.	Erlotinib	Pre	Y	24	8	3	2	1	—	1
P07	F	77	adeno.	Pembrolizumab	Pre	Y	19	9	2.11	0.33	1.77	—	11
P08	F	87	adeno.	Ipilimumab, Nivolumab	Pre	—	16	8	2	0	2	—	93
P09	F	91	adeno.	Ipilimumab, Nivolumab	Pre	—	10	8	1.25	0	1.25	—	18
P10	M	65	adeno.	Ipilimumab, Nivolumab	Pre	—	3	8	0.38	0	0.38	—	11
P11	M	66	adeno.	Ipilimumab, Nivolumab	Pre	—	2	6	0.33	0.16	0.16	—	19
P12	F	60	adeno.	Ipilimumab, Nivolumab	Pre	—	2	7	0.29	0	0.29	—	27
P13	M	65	adeno.	Pembrolizumab	Pre	—	1	6	0.17	0	0.17	—	23
P14	M	64	adeno.	Pembrolizumab	Pre	—	1	9.8	0.1	0.1	0	—	2
P15	F	75	squamous	Ipilimumab, Nivolumab	Pre	—	0	6	0	0	0	—	13
P16-1	M	62	squamous	Avelumab	Pre	—	20	6	3.33	3	0	0.33	10
P16-2					On treatment	—	5	8	0.62	0.5	0.12	—	10
P16-3					On treatment	—	11	6	1.83	1.83	0	—	5.3
P16-4					On treatment	—	31	8	3.87	3.5	0.12	0.25	14.9
P16-5					On treatment	—	6	6	1	1	0	—	10
P17	M	78	adeno.	Pembrolizumab	Pre	—	3	8	0.38	0.125	0.25	—	13
P18	M	82	adeno.	Ipilimumab, Nivolumab	After 1st dose	—	1	6	0.17	0.17	0	—	19
P19-1	M	69	squamous	Pembrolizumab	After 1st dose	—	4	6	0.67	0	0.67	—	9.33
P19-2					On treatment	—	6	8	0.75	0.5	0.25	—	3.75
P19-3					On treatment	—	1	8	0.12	0	0.12	—	17.62
P19-4					On treatment	—	58	6	9.67	1.33	8.33	—	16.83
P19-5					On treatment	—	8	8	1	0.62	0.25	0.12	7.5
P19-6					After treatment	—	1	6	0.17	0.17	0	—	20.5
P20	M	76	adeno.	Pembrolizumab	On treatment	—	43	6	7.16	0.16	6.83	0.16	30
P21	F	51	adeno.	Avelumab	On treatment	—	24	6	4	0.5	3.5	—	20
P22	M	74	squamous	Pembrolizumab	On treatment	—	2	8	0.25	0	0.25	—	5
HD01	F	57	Healthy	—	—	—	2	4	0.5	—	—	—	14
HD02	M	55	Healthy	—	—	—	5	4	1.25	—	—	—	30.75
HD03	M	20	Healthy	—	—	—	0	2	0	—	—	—	18.5
HD04	M	23	Healthy	—	—	—	2	6	0.33	—	—	—	12
HD05	M	35	Healthy	—	—	—	5	6	0.83	—	—	—	19.3
HD06	F	69	Healthy	—	—	—	6	6	1	—	—	—	17.3
HD07	F	77	Healthy	—	—	—	2	6	0.33	—	—	—	27.6
HD08	F	41	Healthy	—	—	—	4	6	0.66	—	—	—	21.3
HD09	M	69	Healthy	—	—	—	1	6	0.16	—	—	—	29
HD10	F	60	Healthy	—	—	—	3	6	0.5	—	—	—	13.83

31 patient blood samples and 10 healthy donor samples were processed through Vortex technology. These 31 samples were collected from 22 different NSCLC patients: 5 squamous, 17 adenocarcinoma, with 17 blood samples collected pre-treatment, 2 after the first cycle, and 11 during the treatment. 15 samples had ≥10 CTCs and were considered for further study, with 4 of them having a biopsy of the primary tumor available. (PDL1+), (PDL1−) and (UD) respectively indicate the number of CTCs per mL that are either identified as PD-L1 positive, PD-L1 negative or undetermined.

## Methods

### Patient cohort and blood donation

This study included 32 volunteers for blood donation, 22 metastatic NSCLC patients and 10 healthy donors (Table 1). Among the 22 patients, 10 NSCLC patients were receiving anti-PD-1 treatment pembrolizumab, 9 NSCLC patients receiving anti-PD-1 treatment nivolumab (Bristol-Myers Squibb), and 2 NSCLC patients receiving anti-PD-L1 treatment avelumab (EMD Serono). 1 patient was evaluated for treatment with pembrolizumab but eventually received erlotinib. For 2 patients (patients #16 and 19), blood was collected at several time points; 5 times for patient 16 (before the treatment, and 4 follow-up draws) and 6 times for patient 19 (after the first dose, 4 follow-up draws, after the treatment).

As this was a pilot study, samples were evaluated at different time points, though effort was made to collect blood samples prior to any treatment commencing or within three weeks of starting treatment. Of these 31 patient samples, 17 were sampled prior to commencing treatment, 2 were sampled after the first dose after two weeks, while the remaining 11 were taken at various time points while on treatment and 1 after the treatment. All patients receiving pembrolizumab were categorized as having positive PD-L1 expression in their original tumor biopsies^7^. 4 matched biopsies were available for analysis for this study.

This study was approved by the UCLA IRB (protocol #11-001798). All patients provided informed consent for participation in this study. After obtaining informed consent, 6–10 cc of blood were collected from each patient in EDTA tubes. Collected samples were processed to isolate CTCs within four hours of collection. Blood samples from healthy volunteers (n = 10) of various ages were similarly processed. All methods were performed in accordance with the relevant UCLA IRB guidelines and regulations.

### Isolation of CTCs using Vortex technology

We used a microfluidic device for rapid, size-based capture of CTCs from blood called the Vortex HT chip, as previously described by the authors^23^ (Fig. 1A). The Vortex HT chip utilizes inertial microfluidic flow to isolate CTCs within microscale vortices. Captured cells are then released and collected off-chip in a well plate for fixation and immunostaining.

### Cells lines and WBCs

Lung cancer cell line staining controls A549 (adenocarcinoma), H1703 (squamous carcinoma) and H3255 (adenocarcinoma) were cultured in RPMI media supplemented with 10% FBS and 1% pen/strep. Hela cells were cultured with DMEM media supplemented with 10% FBS and 1% pen/strep. At 70% confluence, cells were harvested using 0.25% trypsin and fixed with 2% paraformaldehyde. White blood cells (WBC) were isolated from healthy blood using RBC lysis buffer (eBioscience). WBCs were similarly fixed with 2% paraformaldehyde. During each staining experiment, an aliquot of each of these fixed cell solutions was stained in the same well-plate alongside with the CTC samples for normalization.

### Immunofluorescence staining of circulating tumor cells

Collected cells were fixed with 2% paraformaldehyde (Electron Microscopy Sciences) for 10 min, permeabilized with 0.4% v/v Triton X-100 (Research Products International Corp) for 7 min, blocked with 5% goat serum (Invitrogen) for 30 min. To identify CTCs, cells were labelled for 40 minutes at 37 °C with 4,6-diamidino-2-phenylindole (DAPI) (Life Technologies), anti CD45-phycoerythrin (CD45-PE, Clone HI30, BD Biosciences), and a cocktail of primary antibodies to identify cytokeratin (CK) positive cells (Pan-CK clone AE1/AE3, eBioscience, clone CK3-6H5, Miltenyi Biotec, and CK clone CAM5.2, BD Biosciences). To identify PD-L1 levels, cells were also stained with anti-PDL1 antibody (ProSci Inc), A secondary antibody labeled with Alexa Fluor 647 was used as the fluorescent reporter for the PD-L1 antibody. One set of cells consisting of A549, H1703 and healthy white blood cells (WBCs) were stained along with each patient sample. These additional cells were necessary to normalize for the staining process, antibody batch, and microscope conditions over the length of the study and report fluorescent intensities that could be compared between CTCs from many samples. The staining protocol for PDL1 was optimized to positively stain lung cancer cell lines (Supp. Figure 1). After staining, the cells were imaged (Axio Observer Z1, Zeiss) and manually enumerated using specific classification criteria. We identified CTCs in patient samples based on DAPI+/CK+/CD45−, or DAPI+/CK−/CD45−/ along with cytopathological features of malignancy as described previously^23^. All CTC and WBC counts were checked by two independent reviewers (Table 1). PD-L1 expression on these CTCs was quantified using a semi-automated algorithm as described below. Some CTCs could not be evaluated for PD-L1 expression due to the presence of fluorescent debris overlapping all or part of the cells in the PD-L1 Cy-5 channel and thus obscuring the full PD-L1 signal; the PD-L1 status on these cells was thus considered undetermined (identified as “UD” in Table 1).

### Immunohistochemistry of lung tumor biopsies

Immunohistochemistry was performed by the UCLA TPCL Pathology core facility. Briefly, thin tumor sections were cut from paraffin tissue blocks of biopsies obtained prior to treatment (Fig. 1B). The slides were deparaffinized in xylene and re-hydrated through graded ethyl alcohols (100% x3, 95% x2) to distilled water; initially xylene for about 10 min and the remaining treatments for 1 minute each with agitation. Antigen retrieval was performed in a pressure cooker (5 minutes at max temperature) in high pH Tris-EDTA buffer and samples were cooled for 15 minutes at room temperature after pressure returned to atmospheric pressure. The slides were incubated with primary antibody (rabbit monoclonal anti-PDL1 clone SP142, Sina Biological, at 1/200 dilution) for 60 minutes followed by anti-rabbit, horseradish peroxidase polymer (Refine detection kit from Leica) for 15 minutes. The slides were washed with buffer after each of the primary antibody and polymer steps and then incubated with hydrogen peroxide/diaminobenzidine for 10 minutes. Cells were counterstained with hematoxylin. Biopsy tissues were only available for analysis from 4 patients.

### Quantification of PD-L1 levels on CTCs and tumor samples

**Figure 2:**
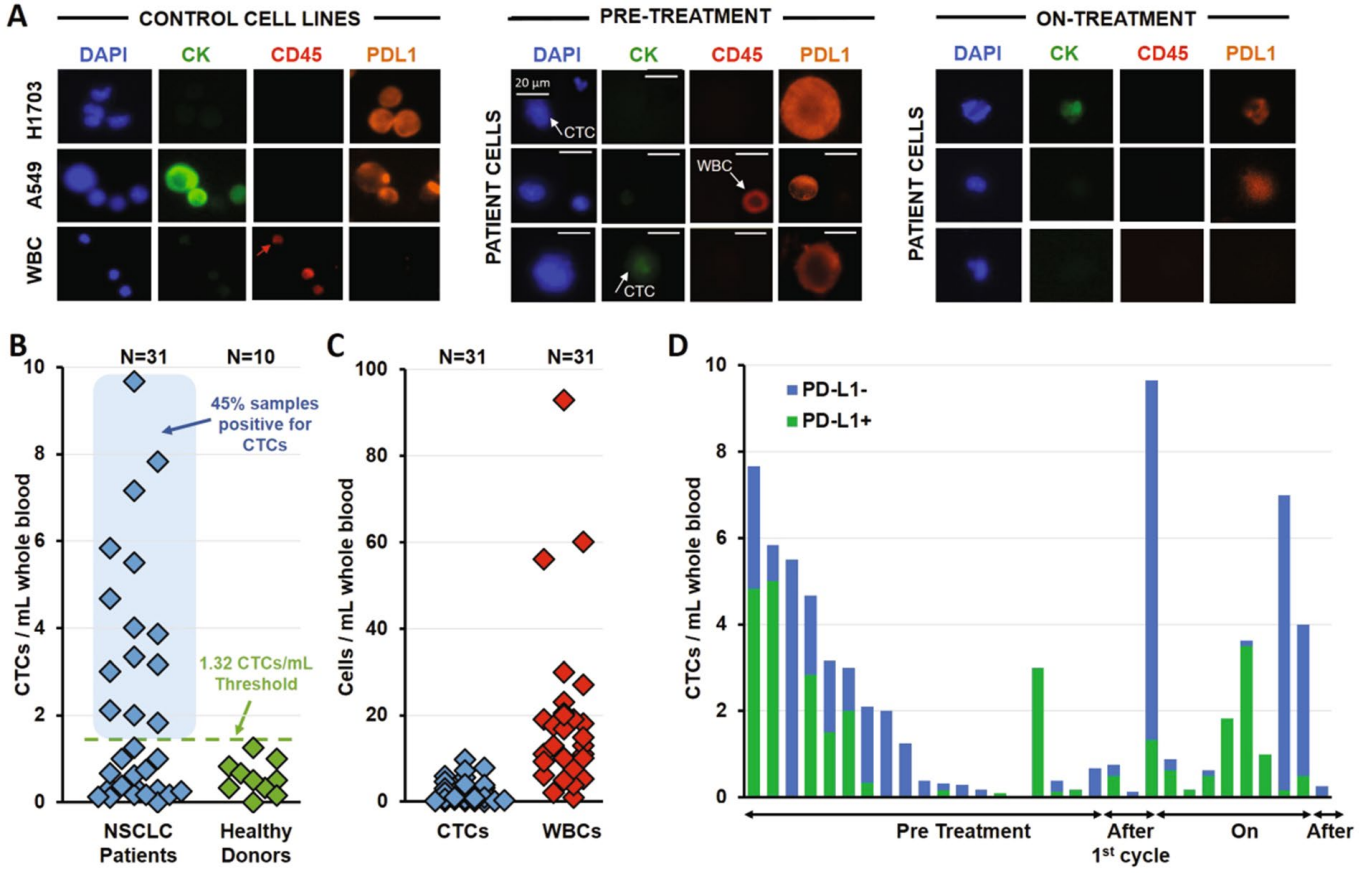
Comparison of PD-L1 expression in CTCs and matched primary tumor prior to anti-PD1 immunotherapy. (**A**) Tumor specimens were analyzed by IHC staining for PD-L1, using DAB Peroxidase (HRP) staining technique. PD-L1 tumor score is the percentage of cells in each category: negative, low, medium, high. CTCs were analyzed by IF and semi-automated quantification of PD-L1 staining intensity (Supp. Figure 1), with CTC intensity normalized by the average staining intensity of H1703 as a control cell line. (**B**) PD-L1 CTC threshold level and IHC PDL1 threshold levels are quantified based on the percentage of cells in four different categories. (**C**) Bar plots showing percentage and PD-L1 level of matched tumor biopsy and CTC specimens (n = 4 patients).

In order to quantify PD-L1 expression on CTCs, we developed a semi-automated imaging algorithm using a custom MATLAB script. The script was used to quantify the cell sizes and normalized fluorescence levels for each cell from each patient sample (Supp. Figure 2). Briefly, an edge detection algorithm was used to locate the outline of the cell membrane (from the transmitted light image) and convert the outline to a binary image mask. The mask was then overlaid against the fluorescence images from each channel and utilized to identify the fluorescence per pixel in each cell. The sum of the pixel intensity of the PD-L1 channel (Alexa Fluor 647) in the area identified as the cell was calculated for each CTC, and ~100 control cells of each type. To normalize the fluorescence intensity of the CTCs, we utilized the lung cancer line H1703 as these cells had the highest expression of PD-L1 of the three lung cancer lines used (A549, H1703, and H3255)^24^. Staining a fixed batch of these cells along with each sample, allowed normalization of the CTC data. We use the following equation (Eq. 1) to calculate the normalized intensity:

1 $$\frac{({\sum }_{n=\,1}^{\#\,of\,pixels}({I}_{pixelofCTC}-local\,background))}{\frac{1}{K}{\sum }_{N=1}^{K}({\sum }_{n=\,1}^{\#\,of\,pixels}({I}_{pixelofaH1703cell}-local\,background))},$$

where *I* is the pixel value and *K* is the total number of H1703 cells analyzed. This value shows the relative intensity of PD-L1 expression on CTCs to that of H1703 cells. If the CTC has much higher expression than H1703, then the normalized value would be greater than 1. Once the CTC fluorescence intensities were normalized, they were categorized as PD-L1 negative (normalized intensity between 0–0.05) or PD-L1 positive. The quantity of PD-L1 expression was further categorized as either low (normalized intensity between 0.05–0.4), medium (0.4–0.7), or high (>0.7) as defined in the cutoff values in Fig. 2B. These descriptor bins were set initially by visual inspection.

To quantify PD-L1 expression on the tumor biopsy sections when available, the thin biopsy specimens were analyzed using the positive pixel count algorithm in HALO software (Indica Labs). The intensity signal from each cell was categorized as negative for PD-L1 expression (intensity between 0–0.04) or as positive (0.04–1). Positive cells were further categorized into low (intensity between 0.04–0.1), medium (0.1–0.2) and high levels (0.2–1), as indicated by the cutoff values shown in Fig. 2B. The lymphocytes at the periphery of the tumor were excluded by the software based on the cell size and nucleus.

### Association with outcome and statistical analysis

We categorized patients by number of CTCs (<1.32 CTCs/mL or ≥1.32 CTCs/mL) and obtained clinical information (progression free survival) and immune related response criteria (irRC) based on imaging scan data when available^25,26^. Patients were categorized by the overall best response to treatment (complete response, partial response, stable disease, progressive disease, or not evaluable). The association between CTC and PD-L1+ counts with progression free survival was performed using Cox proportional-hazards models. In order to quantify the effects of interest, hazard ratios (HR) along with 95% confidence intervals (CI) were estimated by the model. All statistical analyses were performed using R V3.1.2 (Vienna, Austria) and IBM SPSS V23 (Armonk, NY). P values less than 0.05 were considered significant.

## Results

### CTCs can be enriched from NSCLC patient blood samples

We first confirmed that CTCs could be enriched from metastatic NSCLC patients with Vortex technology. A total of 22 patients were enrolled in this study, with 31 samples collected, processed with Vortex technology and enumerated for CTCs. 30/31 (96.8%) samples had at least 1 CTC, 15/31 (48.4%) samples at least 10 CTCs, with CTC total counts ranging from 0.1 to 9.67 CTCs/mL of blood (Table 1, Fig. 2B). Besides the CTCs, between 1 and 93 WBCs were collected per mL, which indicates a high-purity (Table 1, Fig. 2C). As negative controls, we tested blood samples from 10 healthy volunteers, male and female, of different ages (Table 1, Fig. 2B). Using the same enumeration criteria described for the patients, 0 to 1.25 cells per mL were isolated from healthy controls and characterized as CTCs. Based on these enumeration data, a “healthy” cut-off value was defined as the mean number of CTCs + 2 SD (mean = 0.556, SD = 0.385), and thus the cut-off was calculated to be 1.32 CTCs/mL. Using this threshold, 14 of 31 patient samples (45%) were considered positive for CTCs.

### PD-L1 can be quantified on CTCs prior to treatment with PD-1 inhibition

We then developed a method for quantifying PD-L1 expression on lung cancer cells using immunofluorescence staining. To identify the optimal primary and secondary antibody concentrations, we utilized HeLa cells as a positive control and RBCs as a negative staining control for PD-L1 (Lee) We tested three commercially available PD-L1 antibodies (ProSci Ref# 4059, BioLegend clone: 29E.2A3, and eBioscience clone: MIH1) and determined that the ProSci antibody had the most intense specific staining while maintaining the least non-specific staining (Supp. Figure 1A).

We used the three lung cancer cell lines A549, H1703 (adenocarcinoma), and H3255 (squamous cell carcinoma) to develop and validate the PD-L1 fluorescence immunostaining protocol and quantification algorithms. The H1703 line was found to have the highest overall expression of PD-L1 while H3255 had minimal to no PD-L1 expression (Supp. Figure 1B–C); we thus decided to use H1703 as the positive staining control, whereas H3255 were used as the negative staining control.

Once these parameters had been determined, we then implemented this protocol to quantify PD-L1 expression on the isolated lung cancer patient CTCs; with examples of patient sample staining shown in Fig. 2A. For each patient, the number of CTCs positive and negative for PD-L1 were counted (Table 1, Fig. 2D). Of patient samples with CTCs, 30/31 had one or more PD-L1 + CTCs (Fig. 2D). The fraction of PD-L1 positive CTCs among these patients ranged from 2.2 to 100% (Table 1, Fig. 2D).

### PD-L1 expression on tumor biopsy sections can be quantified and compared with CTC expression prior to treatment

**Figure 3:**
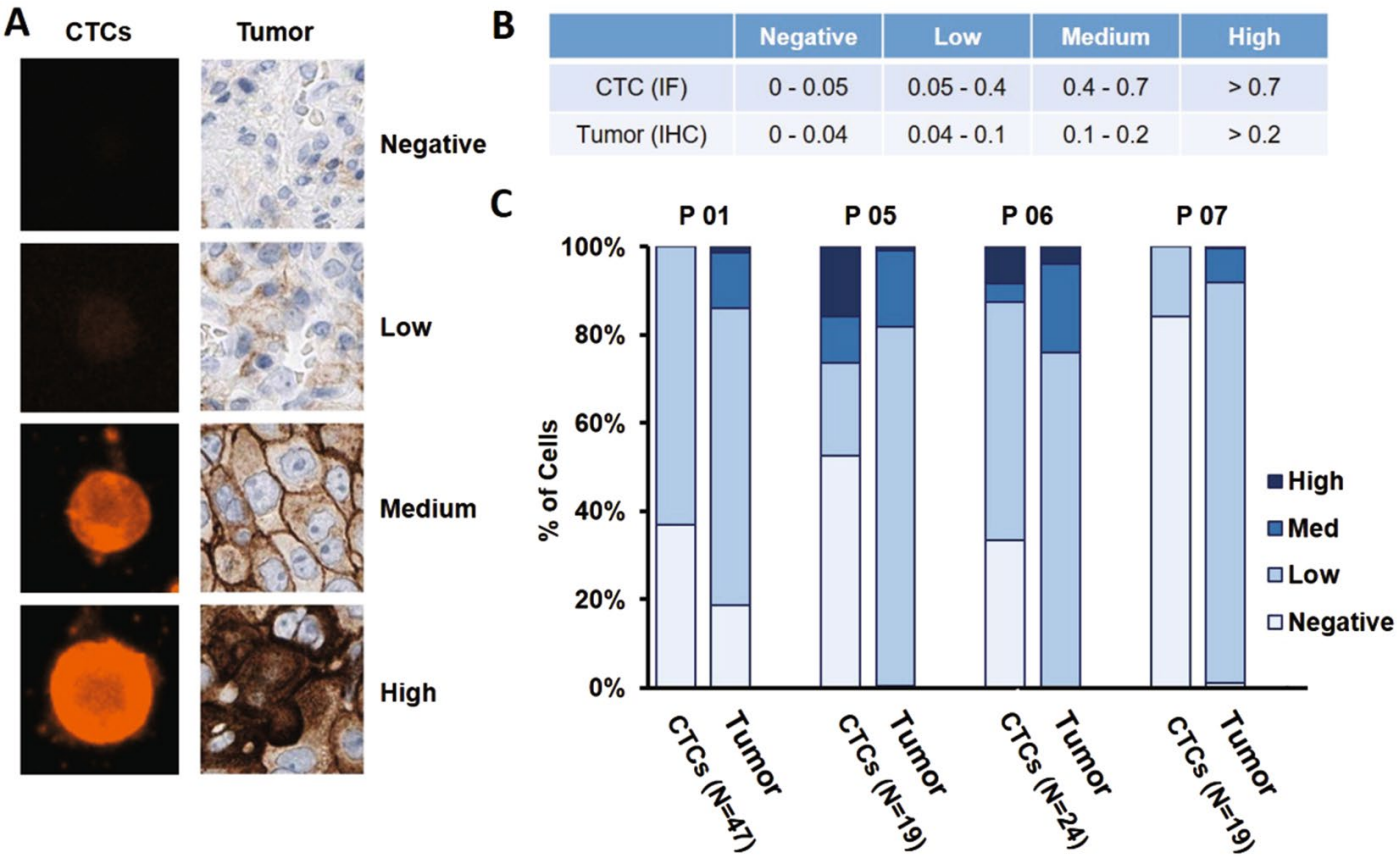
CTC Immunostaining, enumeration, and PD-L1 expression analysis. (**A**) Gallery of cells collected through Vortex technology from patient samples. Healthy WBCs and lung cancer cell lines are used as staining controls. Cells were immunostained for PD-L1-AF647, CK-FITC, CD45-PE and DAPI, then classified according to quantitative criteria previously published^23^. (**B**) Following this method, 10 healthy donors blood samples and 31 samples from 22 patients were processed and CTCs enumerated. A healthy threshold can be defined from the healthy donors as 1.32 CTCs/mL (average + 2 SD). Using such a threshold, 14 of 27 patient samples were positive for CTCs (52%). (**C**) CTCs are collected with high purity, with 0–9.67 CTCs/mL among 1–93 WBCs/mL. (**D**) CTCs captured from different patients at different points in the course of their therapy display varying levels of PD-L1 expression. Each bar represents a distinct patient and a distinct time point.

We next examined the concordance of PD-L1 staining between CTCs and tumor biopsy sections, as PD-L1 positivity in these sections has been shown to be a predictor for outcome in lung cancer and the PD-1 inhibitor pembrolizumab requires a patients’ tumor biopsy to be positive for PD-L1 prior to administration (as determined by an FDA-approved companion diagnostic). Tumor biopsies prior to treatment were only available for 4 patients from the 22 patients in this study. For these 4, thin sections of tumor were cut from the paraffin block, stained and the resulting PD-L1 levels quantified as described above. Sample images for negative, low, medium, and high PD-L1 staining are shown in Fig. 3A. Although all 4 tumor biopsies were initially scored as PD-L1 positive, we quantified PD-L1 levels and found that the majority of positive cells had low expression (Fig. 3C). Patient P07 had the lowest fraction of medium (7.64%) and high (0.46%) staining cells in the corresponding biopsy, with 91.9% of the cells being either low or negative for PD-L1. This was reflected within the CTCs as well, as P07 had the lowest fraction of PD-L1 positive CTCs (15.8%, i.e. 3 of 19, these 3 cells being even identified as low PD-L1 expression). Two patients (P05 and P06) with the highest fraction of positive PD-L1 cells in their tumor (P05: 99.5%, P06: 99.9%) also had the highest fraction of PD-L1 positive CTCs (P05: 47.4%, P06: 66.7%), with a significant part of CTCs having medium or high PD-L1 expression (P05: 26.3%, P06: 12.5%) and (P06: 4 of 24 in P06). For Patient P01, tumor and CTCs provided again a similar pattern, with respectively 18.6% and 37% of the tumor cells and CTCs being PD-L1 negative, while 67.3% and 63% of the cells were PD-L1 positive but with a low expression level. Interestingly, for all 4 patients, the fraction of CTCs that were negative for PD-L1 staining was always higher than the fraction of negative cells in the corresponding biopsy.

### Association of CTC count and progression free survival

**Figure 4:**
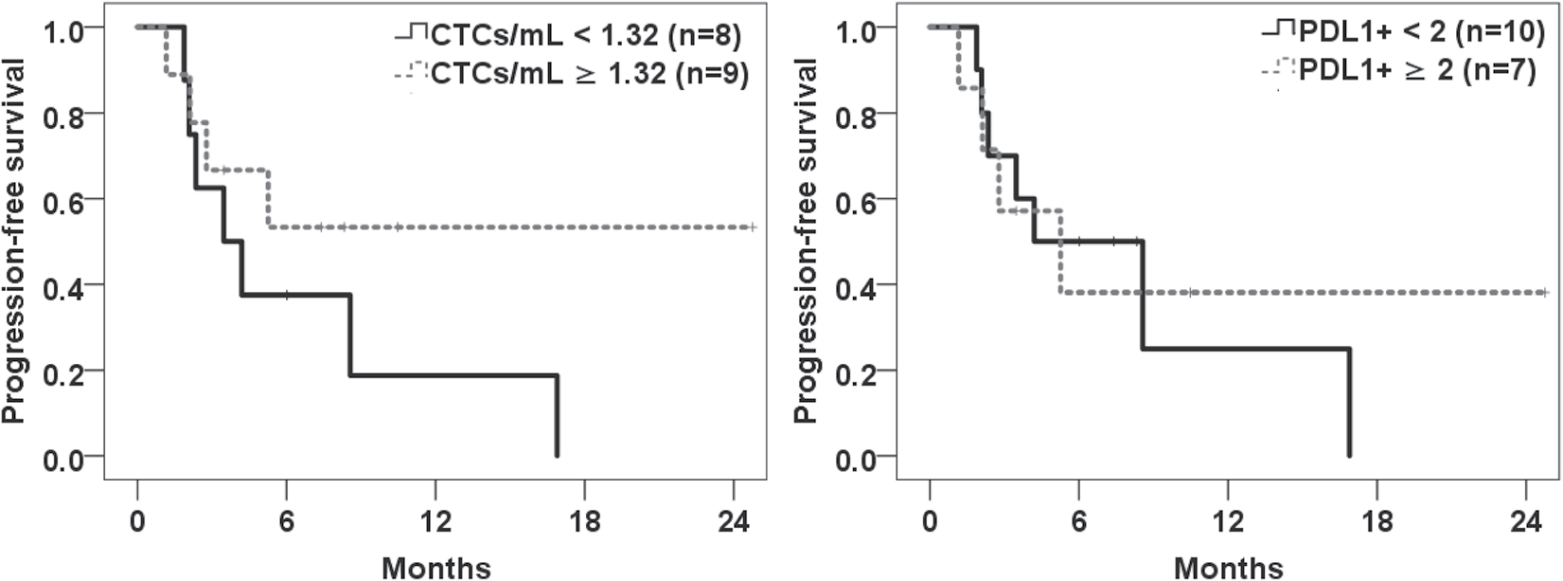
Kaplan-Meier Curves for patients receiving PD-1 inhibition, with collection prior to initiating treatment (n = 17). The curves on the left shows PFS for overall CTC count (< or ≥1.32/mL) while the curves on the right show PFS for PD-L1 + CTCs (total number < or ≥2).

As PD-L1 expression is detectable on CTCs, we wanted to determine whether its detection can be an adjunct predictor for response to PD-1/PD-L1 inhibition. We determined progression using the irRC criteria. To assess the impact of overall CTC count and PD-L1 expression, we counted all CTCs and the subset that were PD-L1 positive (Table 1, Fig. 2D). We limited the analysis for this section to those patients who had a blood collection immediately prior to starting treatment (n = 17). For patients still having response to treatment, the cutoff day for response was set at July 8, 2016. For total CTC count, we pooled patients into two categories: those with <1.32 CTC/mL and those with ≥1.32 CTCs/mL (Fig. 4). Using this categorization, the hazard ratio for progression free survival on PD-1/PD-L1 inhibition was 0.48 (95% CI, 0.14–1.64, P = 0.239) for patients with ≥1.32 CTCs/mL. We also analyzed the association of PD-L1 expression and progression free survival for the 17 patients prior to starting treatment. The hazard ratio for PFS for patients with ≥2 PD-L1 + CTC (overall count) was 0.83 (95% CI, 0.24–2.84, P = 0.764). Of note, of the 5/17 patients with either partial response (PR) or stable disease (SD), three of the five had >50% PD-L1 + CTCs, while the other two had no PD-L1 positive CTCs; however, given the small number of patients with response, this association needs to be analyzed in a larger cohort to determine the overall impact of an increased fraction of PD-L1 + CTCs.

**Figure 5:**
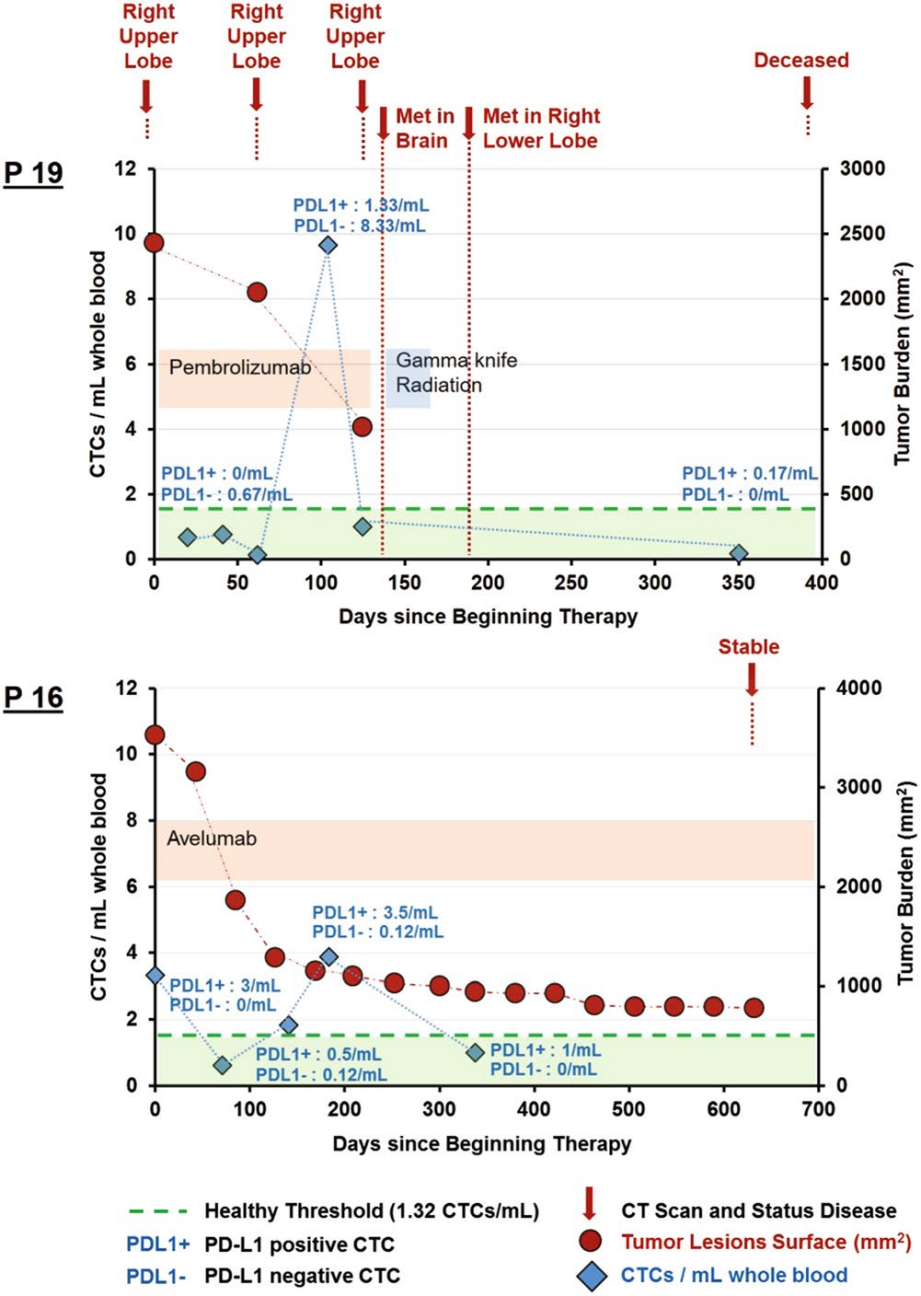
CTC counts during the clinical course and treatment of Patients 16 and 19, since initiation of treatment. Orange and blue bars represent periods when patients were receiving inhibitor therapy, either avelumab or pembrolizumab and radiation respectively. Green dotted line represents the healthy threshold of 1.32 CTCs/mL above which patients are defined as positive for CTCs. Blue marker indicates the CTC number at the time of blood collection, with indication of PD-L1 positive and negative CTCs. The red circle marker indicates the tumor burden, i.e. the index tumor lesions summed cross-sectional area. (Top) In Patient P19, tumor burden initially decreased over time indicating a shrinking of the right upper lobe tumor and a potential response of the patient to the treatment. However, the patient soon after was found on imaging to have developed metastases to the brain and lung, which was preceded by the increase of CTC number from 0.67 to 9.67 CTCs/mL. The patient died at day 538. (Bottom) In Patient P16, tumor burden decreased over time and was stable at the last time point. CTC number varied from 0.62 to 3.87 CTCs/mL but always with a high proportion of the CTCs being PD-L1 positive (between 80.6% and 100%).

Although for this initial study, we did not serially collect blood in all patients, we did so for two patients (Patients #16 and 19, Fig. 5). For Patient P19, the first collection was after the first dose of pembrolizumab (Fig. 5.top). The patient continued to receive pembrolizumab as he was having decrease in tumor burden as measured by radiographic scan. However, a blood draw two and a half months after the first draw revealed an increase in CTC count from 0.67 to 9.67 CTCs/mL, with a low fraction of PD-L1 + CTCs (13.7%) (Table 1). Imaging conducted one month later demonstrated new brain metastases; the patient expired one year later. For Patient P16, 5 blood draws were collected at the beginning and during the course of avelumab therapy. Tumor burden was measured as well and decreased over time. Patient was indicated as stable at the last CT scan. During the 5 blood draws, CTC number varied from 0.62 to 3.87 CTCs/mL, but always with high proportion of the CTCs being PD-L1 positive (between 80.6% and 100%). Interestingly, at the exact beginning of the PD-L1 inhibitor therapy, 100% of the CTCs collected were defined as PD-L1 positive. Future work will involve serially tracking patients to see how CTC monitoring (both total and PD-L1 + CTC level) may correlate with efficacy or loss of efficacy of treatment.

## Discussion

Although immunotherapy represents a breakthrough in the treatment of selected cancers, only a fraction of patients respond. In metastatic NSCLC, the overall response rate is approximately 20%^7,14^. Although several studies have indicated that selected biomarkers (such as tumor PD-L1 expression or the presence of CD8 + infiltrating lymphocytes) are correlated with patient response, there is still a need for other ancillary and non-invasive biomarkers that can be used to monitor response to ultimately help guide the treatment course. CTCs have recently gained momentum as a non-invasive liquid biopsy for monitoring of treatment response and as a source of genetic material to understand treatment failures. Although the analysis of circulating cell-free tumor DNA (ctDNA) is also being explored, especially in identifying the presence of known druggable mutations, such an approach is not suited for identifying phenotypic changes such as levels of PD-L1 on tumor cells. There are no known consistent genetic lesions associated with up-regulated expression of PD-L1. Also, a variety of other cell types express PD-L1 (for example macrophages^13^) and could release protein, extracellular vesicles, or mRNA into the blood stream. CTCs are ideally suited to characterize PD-L1 expression through a non-invasive liquid biopsy in that they are short-lived markers of the active invasive tumors, with holistic phenotypic and proteomic information.

This study represents one of the first to examine PD-L1 expression on NSCLC CTCs in the context of anti-PD1/PD-L1 treatment. Some of the newly approved checkpoint inhibitors require a companion diagnostic indicative of tumor positivity for PD-L1 prior to administration (specifically pembrolizumab). So, in this pilot study, we wished to develop algorithms to quantify PD-L1 expression on CTCs and tumor biopsies respectively and secondly, explore whether CTC PD-L1 expression was correlated with tumor expression. Although PD-L1 expression alone was not predictive of progression free survival, we did note several potential trends in CTC count and PD-L1 expression. Even given a limited sample size, we did note that for patients with >1.32 CTCs/mL, those with >50% PD-L1 + had improved overall response (3 of 4 patients), though most of these patients were also PD-L1+ in their tumor biopsies. Heterogeneity of PD-L1 levels could indicate intra-tumoral or intra-patient heterogeneity, as each of these patients had multiple metastatic sites and the CTCs could break off from any or multiple of these sites.

Nevertheless, the trends we find here are suggestive that PD-L1 status on CTCs may track that of tumor tissue and that this may be a useful correlate in helping to assess potential for response to immunotherapy. Our findings indicate that PD-L1 expression on CTCs can be quantified as an adjunct biomarker. This work also suggests that serial monitoring of patient overall CTC count and PD-L1 + CTC count may help to identify changes in response to treatment, and these cells may be a readily obtainable source to understand tumor evolution, work that needs to now be confirmed with larger prospective studies. Future work will examine the time course of CTCs and their PD-L1 levels during immunotherapy treatment as well as how changes in CTC count and PD-L1 expression may correlate with overall response.

One limitation of this study or any CTC study in general is that it can be difficult to draw associations with few cells. In many cases, CTCs are present prior to treatment but their numbers can be limited, due in part to previous chemotherapeutic treatment or the fundamental patient-to-patient heterogeneity of tumor behavior. This fundamental issue is exacerbated by practical challenges with the limited number of patient samples that are available from patients on clinical trials for new therapies.

## Conclusions

CTC PD-L1 quantified levels, when combined with the tumor biopsy results, could aid in identifying patients more likely to respond to therapy or likely to have become resistant to treatment when tracking levels over time. This study indicates that continued work is warranted to analyze and compare more CTC samples from patients on anti-PD-1 pathway treatment to more robustly determine how PD-L1 expression correlates to tumor levels, fluctuates in response to treatment, and is predictive of response. The methods we describe here are potentially applicable to any tumor type and to potentially any treatment course, as the size-based approach does not exclude cells on the basis of presence or absence of surface markers and the size selectivity criteria^27^ can be tuned based on the cancer type known to be present.

## Electronic supplementary material

Supplementary Figures
