# Supplementary material for: Evaluation of PD-L1 expression on vortex-isolated circulating tumor cells in metastatic lung cancer
Source: Sci Rep. 2018 Feb 7;8:2592. doi: 10.1038/s41598-018-19245-w (PMC5803213; doi:10.1038/s41598-018-19245-w)

Supplementary Information

Evaluation of PD-L1 expression on vortex-isolated circulating tumor cells in metastatic lung cancer

Manjima Dhar, Jessica Wong, James Che, Melissa Matsumoto, Tristan Grogan, David Elashoff, Edward B. Garon, Jonathan W. Goldman, Elodie Sollier-Christen, Dino Di Carlo, Rajan P. Kulkarni

SUPPLEMENTARY FIGURES

Supplementary Figure 1. Optimization of PDL-1 immunostaining. (A) Several antibody brands were first tested with RBCs, WBCs, and HeLa cells: BioLegend, ProSci, and eBioscience. ProSci was chosen as it provided the highest staining intensity. (B, C) Using the optimal conditions of anti-PDL1 (ProSci Inc) at a concentration of 50µg/mL, and goat anti-rabbit Alexa Fluor 647 (Cell Signaling) at a concentration of 2µg/mL, respectively as primary and secondary antibodies, PDL-1 staining was tested on several lung cancer cell lines: A549 (adenocarcinoma), H1703 (adenocarcinoma), H3255 (squamous) and WBCs as a control. (D) Once validated, patient samples were stained for PD-L1, CK, CD45, DAPI. CTCs were identified using a classification criteria previously described (23). For each CTC sample, 2 independent controls were used to confirm the validity of the staining: A549 as positive expression for PD-L1 and CK, WBCs as positive control for CD45.

Supplementary Figure 2. Semi-automated Quantification of Fluorescence. A semi-automated image detection algorithm was developed in MATLAB to quantify the PD-L1 intensity on individual cells. After cells were manually classified as either CTCs or WBCs, their centers were marked. (i) The algorithm used these center coordinates to crop a 80x80 pixel box around the cells, both WBCs and CTCs. (ii) An edge detection algorithm located the outline of the cell membrane (from the transmitted light image) and then made a binary image mask. (iii) The mask overlay on the fluorescence images was used to identify the fluorescence intensity per pixel in each cell. (iv) The local background was subtracted. (v) The sum of all pixel intensity in the PD-L1 channel in the overall area identified as being the cell and control cells of each type was calculated. The final normalized PD-L1 intensity value included the sum of intensity per cell divided by the average integrated intensity for H1703 cells stained in parallel.

Supplementary Figure 1

A

1º	BioLegend (12.5 μg/mL)			ProSci (50 μg/mL)			eBioscience (25 μg/mL)
2º	RBC	WBC	Hela	RBC	WBC	Hela	Hela
2 μg/mL	-	-	++	NA	-	++	-
4 μg/mL	-	-	+	-	+	+	-

B

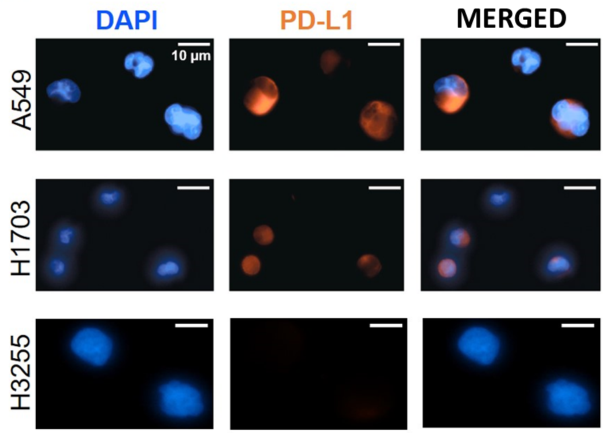

C

1º	ProSci (50 μg/mL)		
2º	A549	H1703	H3255
2 μg/mL	++	++	-

D

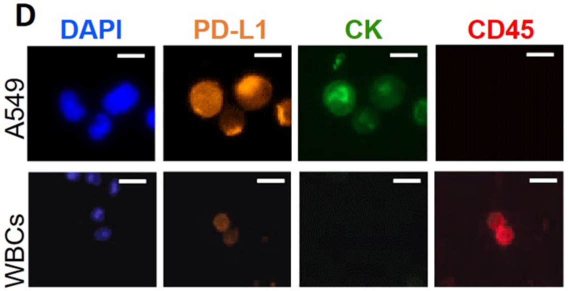

Supplementary Figure 2

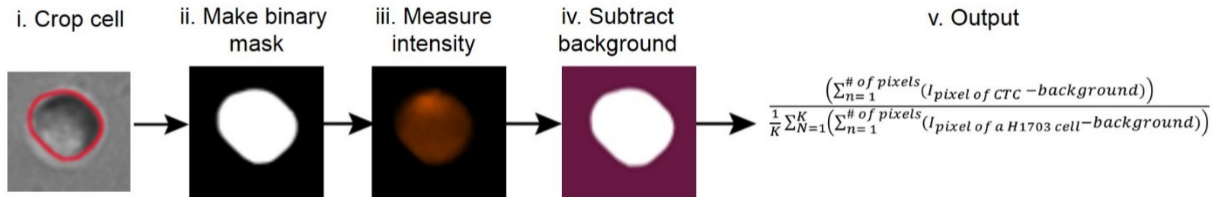

Supplement: Supplementary file 1 — Supplementary Figures [file 41598_2018_19245_MOESM1_ESM.pdf]
